# The Association of Socioeconomic Status (SES) with Procedural Management and Mortality After Percutaneous Coronary Intervention (PCI): An Observational Study from the Pan-London PCI (BCIS) Registry

**DOI:** 10.3390/jcdd12030096

**Published:** 2025-03-10

**Authors:** Krishnaraj S. Rathod, Pitt Lim, Sam Firoozi, Richard Bogle, Ajay K. Jain, Philip A. MacCarthy, Miles C. Dalby, Iqbal S. Malik, Anthony Mathur, James Spratt, Ranil De Silva, Roby Rakhit, Jonathan Hill, Sundeep Singh Kalra, Simon Redwood, Richard Andrew Archbold, Andrew Wragg, Daniel A. Jones

**Affiliations:** 1 Barts Heart Centre, Barts Health NHS Trust, London EC1A 7BE, UK; krishnarajrathod@gmail.com (K.S.R.); ajay.jain1@nhs.net (A.K.J.); a.mathur@qmul.ac.uk (A.M.); andrew.wragg2@nhs.net (A.W.); 2 St George’s Hospital, St George’s University Hospitals NHS Foundation Trust, London SW17 0QT, UK; pitt.lim@stgeorges.nhs.uk (P.L.); s.firoozi@btinternet.com (S.F.); richard.bogle1@nhs.net (R.B.); james.spratt@nhs.net (J.S.); 3 King’s College Hospital, King’s College Hospital NHS Foundation Trust, London SE5 9RS, UK; philip.maccarthy@nhs.net; 4 Harefield Hospital, Guy’s and St Thomas’ NHS Foundation Trust, Uxbridge UB9 6JH, UK; m.dalby@rbht.nhs.uk (M.C.D.); r.desilva@imperial.ac.uk (R.D.S.); j.hill@rbht.nhs.uk (J.H.); 5 Hammersmith Hospital, Imperial College Healthcare NHS Trust, London W2 1NY, UK; iqbal.malik@imperial.nhs.uk; 6 Royal Free Hospital, Royal Free London NHS Foundation Trust, London NW3 2QG, UK; roby.rakhit@nhs.net (R.R.); sundeep.kalra@nhs.net (S.S.K.); 7 St Thomas’ Hospital, Guy’s and St Thomas’ NHS Foundation Trust, London SE1 9RT, UK; simon.redwood@gstt.nhs.uk

**Keywords:** PCI, socioeconomic status, ACS

## Abstract

Background: Lower socioeconomic status (SES) has been associated with increased mortality from coronary heart disease. This excess risk, relative to affluent patients, may be due to a combination of more adverse cardiovascular-risk factors, inequalities in access to cardiac investigations, longer waiting times for cardiac revascularisation and lower use of secondary prevention drugs. We sought to investigate whether socio-economic status influenced long-term all-cause mortality after PCI in a large metropolitan city (London), which serves a population of 11 million people with a mixed social background over a 10-year period. Methods: We conducted an observational cohort study of 123,780 consecutive PCI procedures from the Pan-London (United Kingdom) PCI registry. This data set is collected prospectively and includes all patients treated between January 2005 and December 2015. The database includes PCI performed for stable angina and ACS (ST-elevation myocardial infarction (STEMI), non-ST elevation myocardial infarction (NSTEMI), and unstable angina). Patient socio-economic status was defined by the English Index of Multiple Deprivation (IMD) score, according to residential postcode. Patients were analysed by quintile of IMD score (Q1, least deprived; Q5, most deprived). Median follow-up was 3.7 (IQR: 2.0–5.1) years and the primary outcome was all-cause mortality. Results: The mean age of the patients was 64.3 ± 12.1 years and 25.2% were female. A total of 22.4% of patients were diabetic and 27.3% had a history of previous myocardial infarction. The rates of long-term all-cause mortality increased progressively across quintiles of IMD score, with patients in Q5 showing significantly higher long-term mortality rates compared with patients in Q1 (*p* = 0.0044). This persisted following the inclusion of a propensity score in the proportional hazard model as a covariate (HR for Q5 compared to Q1: 1.15 [95% CI: 1.10–1.42]). Conclusions: This study has demonstrated that low SES is an independent predictor of adverse clinical outcomes following PCI in the large, diverse metropolitan city of London. There clearly are inequalities in cardio-vascular risk factors, time to access to medical treatment/PCI, access to complex imaging and devices during PCI, access to secondary prevention after PCI, and even race differences. Hence, attention to reducing the burden of cardiovascular risk factors and improving primary prevention, particularly in patients with lower SES, is required.

## 1. Introduction

Cardiovascular disease (CVD) remains the leading cause of death in the United Kingdom and worldwide despite significant advances in treatments made in the past century. Lower socioeconomic status (SES) is associated with the development of CVD and appears to convey a risk independent of standard risk factors [1,2]. Those with lower SES not only bear a greater burden of CVD but also appear to have disproportionately worse outcomes especially after acute coronary syndromes (ACS). Data from Denmark found that, even in a country with a universal, tax-financed health care system, patients with lower SES undergoing primary percutaneous coronary intervention (PCI) had worse outcomes than those with more resources [3], findings also replicated in data from Canada [4]. However, other studies have shown that lower SES patients have greater co-morbidities and longer reperfusion times, resulting in comparable outcomes when these are accounted for [5]. This potential excess risk, relative to affluent patients, may be due to a combination of more adverse cardiovascular-risk factors [6], inequalities in access to cardiac investigations [7,8,9], longer waiting times for cardiac revascularisation [10], and lower use of secondary prevention drugs [11,12]. The effect of lower SES on outcome after PCI for coronary artery disease is less well established, with studies both for and against the association [13,14].

### Aims

We therefore investigated to see if SES status influenced procedural outcomes and long-term all-cause mortality after PCI in a large cohort of patients from London, which serves as a diverse population with a mixed social background.

## 2. Methods

We conducted an observational cohort study of 123,780 consecutive PCI procedures from the Pan-London (United Kingdom) PCI registry. This data set is collected prospectively and includes all patients treated by PCI in London, United Kingdom between January 2005 and December 2015. The database includes all patients undergoing PCI performed for stable angina and ACS (ST-elevation myocardial infarction (STEMI), non-ST elevation myocardial infarction (NSTEMI), and unstable angina).

### 2.1. Pan-London PCI Registry

Information about every PCI in the UK procedure is recorded via The UK British Cardiac Intervention Society (BCIS) audit [15]. The database is part of the suite of datasets collected under the auspices of the National Institute for Cardiovascular Outcomes Research (NICOR) and is compliant with UK data protection legislation. Within The Pan-London (United Kingdom) PCI registry, all patients that are treated by PCI in the 9 PCI Centres within London (England, UK) are included. This includes a population of 8.98 million. The nine tertiary cardiac centres in London include Barts Heart Centre (Barts Health NHS Trust), Kings College Hospital (King’s College Hospital NHS Foundation Trust), St Georges Hospital (St Georges Healthcare NHS Foundation Trust), Hammersmith Hospital (Imperial College Healthcare NHS Foundation Trust), Royal Brompton and Harefield Hospitals (Royal Brompton & Harefield NHS Foundation Trust), Guys & St. Thomas’ Hospital (St Thomas’ NHS Foundation Trust), and the Heart Hospital (UCL Hospitals NHS Foundation Trust) Royal Free Hospital (Royal Free NHS Foundation Trust) The registry contains data on 123,780 patients who underwent PCI from 2005 to 2015. The anonymised databases of the 9 London centres that collect data based on the BCIS dataset were merged. The BCIS audit is part of a national mandatory audit that all UK PCI centres participate in. PCI is defined as the use of any coronary device to approach, probe, or cross one or more coronary lesions, with the intention of performing a coronary intervention [15]. At each hospital, there is prospective collection of data. The data are encrypted electronically and then transferred online to a central database. Every patient entry offers details of the patient journey, including the method and timing of admission, inpatient investigations, results, treatment, and outcomes. Information regarding patients’ survival is obtained by linkage of patients’ National Health Service (NHS) numbers to the Office of National Statistics (ONS), which records live/death status and the date of death for all deceased patients. At the time of the procedure and during the admission, patient and procedural details were recorded into the individual centre’s local BCIS database. Anonymous datasets with linked mortality data from the ONS were merged for analysis from the 9 centres.

### 2.2. Study Population and Procedures

We collected patient demographic characteristics including age, left ventricular function, smoking status, previous myocardial infarction (MI), previous revascularisation (PCI and Coronary Artery Bypass Grafting), New York Heart Association classification, and indications for PCI. Further data included presence of hypercholesterolemia, hypertension, cardiogenic shock, diabetes mellitus, pre-procedural cardiac arrest, peripheral vascular disease (PVD) and chronic kidney disease (CKD, defined as Creatinine > 200 micromol/L, or renal replacement therapy). We also collected technical aspects of the PCI procedure and adverse outcomes, including complications up to the time of hospital discharge. All patients undergoing PCI were loaded with either clopidogrel (300–600 mg), ticagrelor (180 mg), prasugrel (60 mg), or aspirin (300 mg) prior to their PCI procedures. The P2Y12 inhibitor was typically continued for 1 year if they had a DES implanted or if they had PCI for an MI, or 1 month if they had a BMS inserted. It was to the discretion of the interventional cardiologist performing the procedure to decide whether adjunctive pharmacology (GPIIb/IIIa inhibitors, bivalirudin, heparin, and thrombolysis) was required for the procedure. Coronary artery disease was classified by severity of luminal narrowing (0%, 1–49%, 50–74%, 75–94%, 95–99%, or 100%) and by vessel affected (e.g., left anterior descending).

### 2.3. Socio-Economic Status

Using the residential postal code, routinely collected as part of the dataset for the 2010 version of the English Index of Multiple Depravation (IMD) Score [16], the socio-economic status of each patient was calculated. The IMD Score is a vigorous index of deprivation which divides England into 32,482 small geographical areas, each of which contains about 1500 residents, and grants them a score for seven domains (health and disability, income, education and training, employment, housing and services, crime, and living environment) according to information obtained from the 2010 national census. Each of the domains were weighted and then pooled to provide a single measure of deprivation for each geographical area. There have been a number of studies that have used IMD scores to investigate relationships between socio-economic factors and health outcomes, such as disease presentation [17], life expectancy [18], equity of access to care [19], and post-surgical mortality [18].

### 2.4. Clinical Outcomes

All data were entered prospectively into an electronic database at the time of the PCI procedure. The data included patient characteristics, procedural details, and complications. The primary outcome was all-cause mortality assessed at a median follow-up of 3.7 (IQR: 2.0–5.1) years. Procedural complications and major adverse cardiac events (MACE) were again recorded prospectively. MACE events were defined as death, MI, and repeat target vessel revascularisation. All-cause mortality status was recorded as of the 10th of August, 2018, and obtained from the British Cardiovascular Intervention Society (BCIS) national database, part of the National Institute of Cardiovascular Outcomes Research (NICOR). This national database is linked periodically to the UK Office of National Statistics and provides the life/death status of treated patients.

### 2.5. Ethics

The data collected were part of a mandatory national cardiac audit and all patient identifiable fields were removed prior to merging of the datasets and analysis. The local ethics committee advised that formal ethical approval was not required for this study.

### 2.6. Statistical Analysis

English IMD score was used to analyse patients by quintile [11]. We used Pearson’s chi-square test for categorical variables and ANOVA for continuous variables for comparison of clinical characteristics of patients. The Shapiro–Wilk test was used to assess normality of distribution. The log-rank test was used to assess survival differences between quintiles. For the effect of socioeconomic status on clinical outcomes in age-adjusted and multiply adjusted models, Cox regression analysis was (hazard ratios (HRs) with 95% confidence intervals (CIs)). This incorporated all available covariates. The proportional hazards assumption was evaluated by examining log (-log) survival curves and tested with Schoenfeld’s residuals. The proportional hazards assumption was satisfied for all outcomes evaluated. A non-parsimonious logistic regression model comparing patients ranked by IMD score propensity score analysis was performed using a statistical program. A number of variables were included in the model, containing all variables with significant interactions. The multivariable Cox regression analysis was performed using only the two variables “propensity score” and “SES” in order to avoid over-adjustment. Statistical analyses were performed using Stata version 14 (Stata Corp, College Station, TX, USA).

## 3. Results

**Figure 1:**
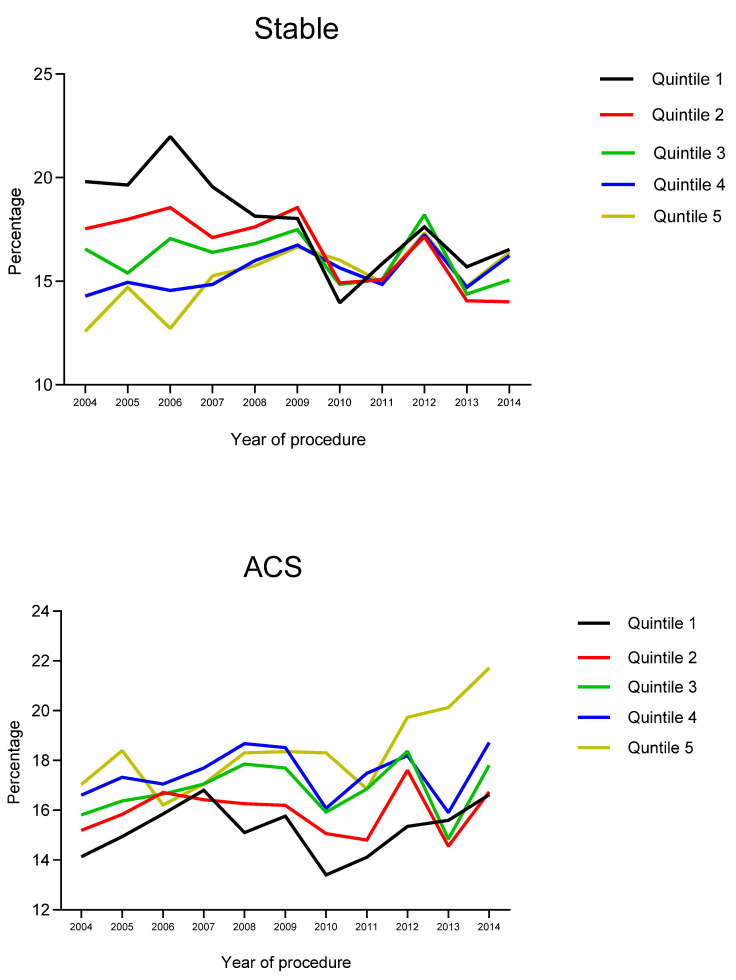
Prevalence of patients in all the quintiles of Index of Multiple Deprivation (IMD). Most-deprived group (Q5) and the least-deprived group (Q1) in both stable and ACS patients.

Overall, there were 123,780 PCI procedures performed during the whole study period. The mean age of the patients was 64.3 ± 12.1 years, and 25.2% were female. A total of 22.4% of patients were diabetic and 27.3% had a history of previous myocardial infarction. The median IMD score was 24.4 (range 13.4 to 38.4). Over the study period, there was an increase in the proportion of patients in Quintile 5 compared to Quintile 1 (Figure 1).

### 3.1. Baseline Characteristics

In the most-deprived group (Q5), patients were significantly younger compared to the least-deprived (Q1) patients and were more likely to be of South Asian ethnicity. In addition, patients in Q5 had higher rates of chronic renal failure (CKD), diabetes mellitus, history of smoking, hypertension, previous MI, peripheral vascular disease, and impaired systolic left ventricular function. However, patients in Q5 had lower rates of previous revascularization (PCI and CABG) (Table 1).

### 3.2. Procedural Characteristics

Acute coronary syndrome, particularly NSTEMI, was the most frequent indication for PCI in patients in Q5 compared with Q1. Furthermore, patients in Q5 were more likely to have their procedure via the radial access for PCI and to have received a GP IIb/IIIa inhibitor and less likely to have a CTO procedure. Patients in Q5 were also less likely to have undergone multivessel PCI or adjunctive intravascular imaging or have a drug-eluting stent inserted (Table 2). Despite the majority of patients being treated with clopidogrel during the study period, following the adoption of newer P2Y12 inhibitors, higher rates of use were seen in Q1 vs. Q5 (of either ticagrelor or prasugrel [53.5% vs. 21.2%]).

### 3.3. Procedural Outcomes

There were no significant differences in procedural success or complication rates between the groups (Q1 to Q5). In-hospital death rates were higher from Q1 to Q5 (Table 3). Length of stay was longer from Q1 (3.6 [IQR: 2.3–6.5 days]) to Q5 {4.9 [IQR: 3.4 vs. 7.3 days]). In addition, bleeding rates were lower from Q1 to Q5.

### 3.4. Long-Term Outcomes

**Figure 2:**
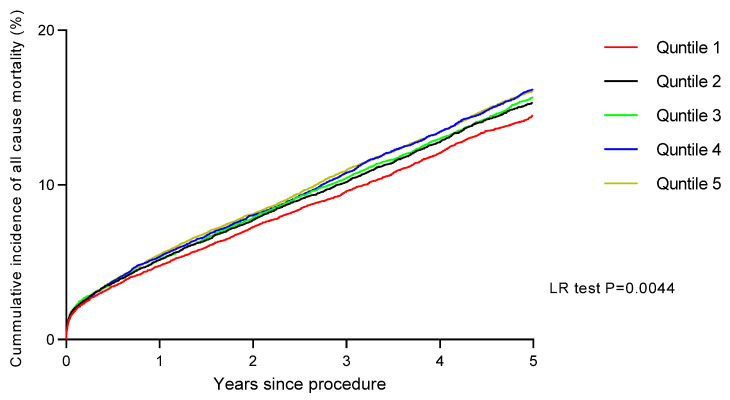
Cumulative incidence of all-cause mortality after PCI by quintiles of socioeconomic status.

**Figure 3:**
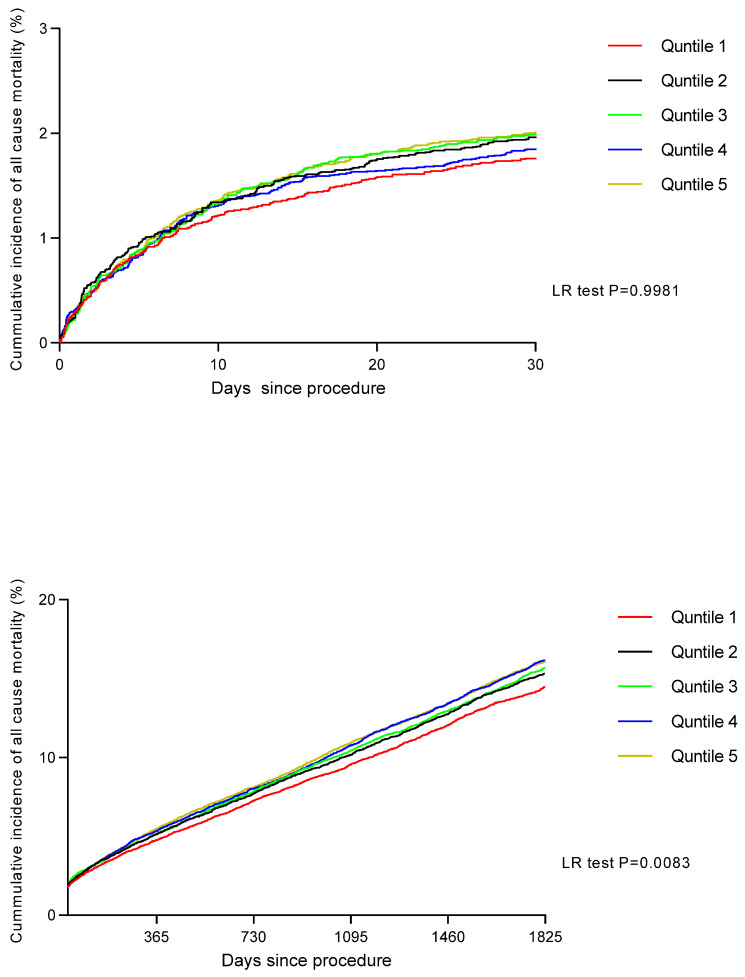
Landmark analysis of cumulative incidence of all-cause mortality after PCI by quintiles of socioeconomic status between 0 and 30 days and then >30 days.

The Kaplan–Meier estimates demonstrated that rates of long-term all-cause mortality increased progressively across quintiles of IMD score, with patients in Q5 showing significantly higher long-term mortality rates compared with patients in Q1 (*p* = 0.0044) (Figure 2). Further landmark analysis demonstrated that the higher long-term mortality rates in Q5 appear to occur after 30 days (*p* = −0.0083) (Figure 3). We also found that age-adjusted HRs for all-cause mortality were significantly higher in patients in Q5, Q4, Q3, and Q2 compared with patients in Q1. In addition, the age-adjusted HR for death was 1.37 (95% CI: 1.18–2.13) for Q5 compared with Q1 (Table 4). Following these multiple adjustments for confounding variables, the HR for death increased (compared to the age-adjusted hazard) for Q5 compared to Q1, at 1.13 (95% CI: 1.07–1.32) (Table 5). We also found that the HRs for death increased in a linear trend for each decreasing quintile of SES. Finally, IMD was associated with long-term mortality following the inclusion of a propensity score in the proportional hazard model as a covariate (HR for Q5 compared to Q1: 1.15 [95% CI: 1.10–1.42]).

## 4. Discussion

This study is the largest study to date evaluating the impact of SES on all-cause mortality following PCI in a large contemporary dataset of nearly 125,000 patients. Patients with lower SES tender to be younger, were more likely to be non-Caucasian in origin and present acutely (ACS), and were less likely to receive guideline-based treatments (i.e., IVUS, DES, procedures, newer P2Y12 inhibitors). Over the study follow-up, despite correction for confounding variables and co-morbidities, lower SES remained associated with a poorer outcome. These findings suggest that SES may have a measurable and significant impact on cardiovascular outcomes after invasive treatment for CAD, with current risk models not adequately accounting for the risk conveyed by lower SES. There is an urgent need to address these inequalities.

This data are comparable to other studies that have shown that SES appears to be independently associated with poorer health outcomes [3,14,19]; however, there are limited long-term data looking at outcome post-PCI. Molendina et al. [4] demonstrated that low SES was associated with increased mortality post-AMI, a finding most pronounced in the short-term but demonstrating these same trends after 1 year post-infarct. In addition, their study showed that in Canada’s universal health care system, there was evidence of reduced access to standard-of-care interventions post-AMI, including cardiac catheterization, revascularization, and rehabilitation, for low-SES patients, highlighting the additional utility of the data provided by our analysis.

Other previous studies have observed that any SES-related differences in clinical outcome can be either partially [7,20,21,22] or completely [7,23] attributed to differences in baseline patient characteristics, which is at odds with our and others’ data suggesting that SES is independently associated with a worse outcome. A number of studies have demonstrated that the patients in lower SES have higher burden of cardiovascular risk factors [3,24,25,26]. At both a community level and at an individual level, SES has been demonstrated to be associated with worse risk factor profile and cardiovascular disease [27]. There have been a few suggestions as to why this may be. Lower levels of participation in screening programs and regular monitoring for multiple disease conditions, including for cardiovascular risk factors, can result in a socioeconomic disadvantage [28,29]. Furthermore, there are lifestyle factors such as lower physical fitness and higher rates of smoking which have been associated with low community SES [30]. Additionally, poverty has been associated with lower use of invasive cardiac procedures in patients with AMI [31]. Kahn et al. [32] identified poorer processes of care in Medicare patients hospitalized with AMI. One of the main advantages of this study is that there is equivalence of insurance for the groups due to the National Health System. Hence, there are no discrepancies in the ‘opportunity’ to access towards healthcare throughout the United Kingdom. An important aspect to note is that the differences in mortality appear to occur almost immediately following PCI; this strongly suggests that the ‘baseline’ differences in SES are likely to contribute significantly to this poorer outcome in the lower-SES groups.

The reasons for worse post-PCI outcomes with lower SES are likely to be multifactorial. A number of studies after AMI have suggested that high-SES patients are more likely to receive guideline-recommended medications at discharge than are low-SES patients [21,33]. In addition, other studies have also demonstrated that patients from low-income backgrounds were less likely to receive secondary medical prevention after 3 months [34] and that discontinuation of evidence-based medication was associated with not graduating from high school [35]. This latter study also suggested that medication therapy discontinuation was associated with higher mortality. However, there have not been any studies that have looked at SES-related differences in clinical outcomes after STEMI that have included information about secondary medical prevention. Hence, we do not yet know if the reported SES-related differences in clinical outcome could be mediated by differences in the secondary medical prevention use during follow-up. The likelihood of a patient taking up and completing a programme of cardiac rehabilitation is strongly influenced by SES, with only 40% of patients from areas of high deprivation (lowest IMD quintile) starting, compared to 54% from areas of low deprivation (highest IMD quintile). Inequalities in access are also seen between gender (71% of those accessing CR in England are male) and ethnicity profiles (over 80% accessing CR in England are White British) (https://www.bhf.org.uk/informationsupport/publications/statistics/national-audit-of-cardiac-rehabilitation-quality-and-outcomes-report-2018 (accessed 10 March 2024)). These findings all represent points at which interventions could be designed to improve access to care for patients with low SES to improve their health and life expectancy after PCI. A study in England determined that CHD mortality was decreasing in individuals of all SES, but the rate of decline was steepest in the most affluent group in comparison with those with lower SES [36]. Hence, there is a need to increase awareness about primary prevention and improve access to primary care services, particularly in communities with high socioeconomic deprivation, to reduce the burden of cardiovascular disease in the future [28,29]. Furthermore, our study also found that patients from the highest SES quintile (Q5) had differences in treatment compared to the patients from the first quintile (Q1) (i.e., lower frequency of DES use, lower use of “newer” P2Y12 receptor antagonists—a known factor for better survival). They were also different in terms of ethnic origin. This has already been described in a previous study by Kolden et al. [37], where ancestry is known to be a significant factor influencing CV morbidity and outcome.

## 5. Limitations

This study is an observational analysis of consecutive patients from a single centre in London. Although our database contained the majority of clinical variables that are known to have an impact on outcome, our result may still be confounded by variables that were not measured (similar to other observational studies). These factors may include cardiac rehabilitation and the impact of secondary medications. However, all data were prospectively collected, and the study’s observational nature meant that the results are reflective of routine clinical practice. Although very robust, the English IMD score has a number of limitations due to the methodology involved in its derivation. The score incorporates seven domains into an overall quantification of deprivation, which is assigned based on defined geographical area rather than on an individual subject’s characteristics. Individuals who live in one particular area will obviously experience different levels of deprivation [37]. IMD scores are not a linear measure of deprivation and do not incorporate information on duration of residence. Therefore, we could not assess the contribution of deprivation exposure time to mortality. Nevertheless, the IMD score is the best available means for quantifying deprivation in England [38]. Another limitation to note is that while the IMD is still the most commonly used small-area metric, by identifying homes in need, other measures (such as the Census household deprivation indicators) can provide further detail to the picture. This is useful in rural areas since the IMD may miss minor deprivation hotspots. Finally, since we do not have secondary prevention data, we cannot comment on the impact of secondary prevention in our patients and how this affects long-term outcome.

## 6. Future Perspectives

There are chances to reevaluate traditional healthcare and offer complimentary treatments at no cost or at a reduced cost. Although clinical settings are typically thought of as the places where healthcare is provided, older persons can also benefit from other community-based resources, services, and initiatives. For instance, in order to assist low-income and vulnerable older persons in improving their health outcomes (such as fall prevention and chronic illness self-management), the federal government of the United States has funded a number of evidence-based initiatives [39,40,41]. These initiatives go beyond conventional clinical settings to enhance healthcare delivery and broaden patient access to medical services.

Reducing health inequities will necessitate a cooperative, multilevel strategy. The guiding ideas include identifying the most vulnerable people and communities and allocating additional resources to them, as well as enhancing cultural competence, expanding access to high-quality healthcare, and modernising medical education. The eradication of health disparities is a crucial task for the federal, local, and corporate governments, as well as employers, healthcare systems, educational institutions, community organisations, and individuals and families. Targeted preschool and early childhood treatments have significant implications for reducing gaps since childhood conditions impact the foundation of SES and health in adulthood [42].

## 7. Conclusions

This study has demonstrated that low SES, as assessed by English IMD, is an independent predictor of adverse clinical outcomes following PCI for in the large, diverse metropolitan city of London. Reducing disparities in health is a major public health challenge, and it is troublesome to see that patients with low SES appear to be significantly disadvantaged with regard to their outcomes following PCI. Hence, attention to reducing the burden of cardiovascular risk factors and improving primary prevention, particularly in patients with low SES, is required.

## Figures and Tables

**Table 1:** Baseline characteristics according to socio-economic status quintile.

	Quintile 1	Quintile 2	Quintile 3	Quintile 4	Quintile 5	*p* Value
	(n = 18,727)	(n = 18,724)	(n = 18,708)	(n = 18,684)	(n = 18,717)	
Age (yrs)	66.70 ± 11.45	65.55 ± 11.86	64.46 ± 12.17	63.14 ± 12.24	64.32 ± 12.15	<0.0001
Ethnicity (Caucasian)	11,817 (63.1%)	10,598 (56.6%)	9092 (48.6%)	7997 (42.8%)	6420 (34.3%)	<0.0001
Gender (male)	14,008 (74.8%)	14,024 (74.9%)	13,975 (74.7%)	13,452 (74.2%)	13,925 (74.4%)	0.358
Previous MI	3521 (18.8%)	3876 (20.7%)	3779 (20.2%)	3774 (20.2%)	3762 (20.1%)	0.218
Previous CABG	4232 (22.6%)	4101 (21.9%)	3816 (20.4%)	3606 (19.3%)	3332 (17.8%)	<0.0001
Previous PCI	5262 (28.1%)	5149 (27.5%)	4958 (26.5%)	4914 (26.3%)	4567 (24.4%)	<0.0001
Hypercholesterolaemia	10,993 (58.7%)	10,879 (58.1%)	10,757 (57.7%)	10,818 (57.9%)	10,294 (55.0%)	<0.0001
Diabetes mellitus	3071 (16.4%)	3782 (20.2%)	4434 (23.7%)	5007 (26.8%)	5615 (30.0%)	<0.0001
Hypertension	10,487 (56.0%)	10,617 (56.7%)	10,907 (58.3%)	11,080 (59.3%)	11,062 (59.1%)	<0.0001
Smoking history	10,244 (54.7%)	10,785 (57.6%)	10,982 (58.7%)	11,715 (62.7%)	12,072 (64.5%)	<0.0001
PVD	543 (2.9%)	618 (3.3%)	617 (3.3%)	654 (3.5%)	674 (3.6%)	0.002
CKD (Creat > 200)	581 (3.1%)	749 (4.0%)	842 (4.5%)	916 (4.9%)	880 (4.7%)	<0.0001
Previous CVA	450 (2.4%)	487 (2.6%)	468 (2.5%)	467 (2.5%)	487 (2.6%)	0.894
Poor LV function	974 (5.2%)	1723 (9.2%)	2077 (11.1%)	1756 (9.4%)	2059 (11.0%)	<0.0001
Cardiogenic shock	412 (2.2%)	431 (2.3%)	468 (2.5%)	486 (2.6%)	468 (2.5%)	0.120

Abbreviations: MI = myocardial infarction, CABG = coronary artery bypass grafting, PVD = peripheral vascular disease, CKD = chronic kidney disease, LV = left ventricular, CVA = cerebrovascular accident.

**Table 2:** Procedural characteristics according to socio-economic status quintile.

	Quintile 1	Quintile 2	Quintile 3	Quintile 4	Quintile 5	*p* Value
	(n = 18,727)	(n = 18,724)	(n = 18,708)	(n = 18,684)	(n = 18,717)	
Access for PCI						
Radial	9832 (25.2%)	4831 (25.8%)	5126 (27.4%)	5400 (28.9%)	5933 (31.7%)	<0.0001
Acute coronary syndrome						
Primary PCI for STEMI	4345 (23.2%)	4494 (24.0%)	4602 (24.6%)	4652 (24.9%)	4773 (25.5%)	<0.0001
PCI for NSTEMI/UA	4700 (25.1%)	5224 (27.9%)	5519 (29.5%)	6035 (32.3%)	6382 (34.1%)	<0.0001
Elective	9289 (49.6%)	8688 (46.4%)	8213 (43.9%)	7623 (40.8%)	7637 (40.8%)	<0.0001
CTOs	1891 (10.1%)	1741 (9.3%)	1702 (9.1%)	1682 (9.0%)	1479 (7.9%)	<0.0001
Left main coronary artery	787 (4.2%)	730 (3.9%)	655 (3.5%)	673 (3.6%)	543 (2.9%)	<0.0001
Right coronary artery	6798 (36.3%)	6853 (36.6%)	7053 (37.7%)	7119 (38.1%)	6963 (37.2%)	0.003
Left anterior descending artery	9326 (49.8%)	9175 (49.0%)	8999 (48.1%)	9024 (48.3%)	9096 (48.6%)	0.020
Left circumflex artery	4607 (24.6%)	4775 (25.5%)	4752 (25.4%)	4820 (25.8%)	4923 (26.3%)	0.004
Vein graft						
Multi-vessel PCI	3521 (18.8%)	3632 (19.4%)	3891 (20.8%)	4017 (21.5%)	4080 (21.8%)	0.042
IVUS use	1854 (9.9%)	1760 (9.4%)	1721 (9.2%)	1607 (8.6%)	1348 (7.2%)	<0.0001
DES use	17,060 (91.1%)	17,001 (90.8%)	16,912 (90.4%)	16,760 (89.7%)	16,827 (89.9%)	<0.0001
GP IIb/IIIa inhibitor	4700 (25.1%)	4999 (26.7%)	5145 (27.5%)	5213 (27.9%)	5634 (30.1%)	<0.0001
Procedural success	18,259 (97.5%)	18,237 (97.4%)	18,259 (97.6%)	18,217 (97.5%)	18,237 (97.4%)	0.215

Abbreviations: DES = drug-eluting stent, IVUS = intravascular ultrasound, RCA = right coronary artery, CTO = chronic total occlusion.

**Table 3:** Procedural outcomes following percutaneous coronary intervention according to socio-economic status quintile.

	Quintile 1	Quintile 2	Quintile 3	Quintile 4	Quintile 5	*p* Value
	(n = 18,727)	(n = 18,724)	(n = 18,708)	(n = 18,684)	(n = 18,717)	
MACE						
Death	206 (1.1%)	243 (1.3%)	243 (1.3%)	280 (1.5%)	299 (1.6%)	0.048
Q wave MI	75 (0.4%)	56 (0.3%)	94 (0.5%)	75 (0.4%)	94 (0.5%)	0.105
Re-Intervention PCI	97 (0.5%)	94 (0.5%)	75 (0.4%)	56 (0.3%)	75 (0.4%)	0.089
CVA	19 (0.1%)	19 (0.1%)	0 (0.0%)	19 (0.1%)	19 (0.1%)	0.125
Elective CABG	37 (0.2%)	19 (0.1%)	19 (0.1%)	19 (0.1%)	19 (0.1%)	0.101
Emergency CABG	19 (0.1%)	19 (0.1%)	19 (0.1%)	19 (0.1%)	19 (0.1%)	0.201
Bleeding	150 (0.8%)	169 (0.9%)	150 (0.8%)	131 (0.7%)	112 (0.6%)	0.027

**Table 4:** Age-adjusted hazard ratios for all-cause mortality after PCI. Age-adjusted hazard ratios of the Cox analysis for all-cause mortality after PCI with 95% confidence intervals.

Variable	Comparator	Age-Adjusted HR	95%CI
Age	Age	1.076	1.074–1.078
Female	Male	0.770	0.644–1.197
Ethnicity (Asian)	Caucasian	1.182	0.945–1.220
Cardiogenic shock	No cardiogenic shock	4.643	4.329–4.981
Smoking history	No smoking history	1.036	0.997–1.076
Diabetic	Non-diabetic	1.528	1.473–1.586
Previous MI	No previous MI	1.492	0.839–1.546
Previous PCI	No previous PCI	1.106	0.765–1.149
Previous CABG	No previous CABG	1.666	0.992–1.744
Hypertension	No hypertension	1.403	1.354–1.453
Hypercholesterolaemia	No hypercholesterolaemia	1.013	0.957–1.049
Previous CVA	No previous CVA	2.887	1.935–4.309
Peripheral vascular disease	No peripheral vascular disease	2.934	2.750–3.131
eGFR < 60 mL/min/1.73 m^2^	eGFR > 60	2.605	2.215–3.064
EF < 35%	EF > 35%	2.179	2.042–2.325
GP IIb/IIIa inhibitor use	No GP IIb/IIIa inhibitor use	0.905	0872–0.940
Procedural success	Procedural failure	0.626	0.583–0.673
Access route (radial)	Femoral	0.880	0.844–0.917
Acute coronary syndrome	Elective procedure	1.209	1.164–1.255
Chronic total occlusions	No chronic total occlusions	1.043	0.987–1.103
Drug-eluting stent use	Bare metal stent use	0.773	0.735–0.812
Multivessel disease	Single vessel disease	1.428	1.380–1.478
Socio-economic status		1.001	1.000–1.012
Socio-economic quintile 2	Socio-economic quintile 1	1.315	1.135–1.523
Socio-economic quintile 3	Socio-economic quintile 1	1.236	1.017–1.502
Socio-economic quintile 4	Socio-economic quintile 1	1.260	1.054–1.664
Socio-economic quintile 5	Socio-economic quintile 1	1.367	1.177–2.130

CABG: coronary artery bypass grafting; CVA: cerebrovascular accident; MI: myocardial infarction; PCI: percutaneous coronary intervention; PVD: peripheral vascular disease.

**Table 5:** Multivariate hazard ratios for all-cause mortality after PCI. Multivariate hazard ratios of the Cox analysis for all-cause mortality after PCI with 95% confidence intervals.

**Variable**	**Comparator**	**Age-Adjusted HR**	**95%CI**
Socio-economic status		1.001	1.000–1.002
Socio-economic quintile 2	Socio-economic quintile 1	1.080	1.023–1.140
Socio-economic quintile 3	Socio-economic quintile 1	1.089	1.017–1.167
Socio-economic quintile 4	Socio-economic quintile 1	1.124	1.021–1.237
Socio-economic quintile 5	Socio-economic quintile 1	1.130	1.070–1.316

## Data Availability

The data is no available due to privacy.
